# Subcultural Differences in Processing Social and Non-social Positive Emotions Between Han and Uygur Chinese: An ERP Study

**DOI:** 10.3389/fpsyg.2019.02041

**Published:** 2019-09-06

**Authors:** Xinmei Deng, Yuanyuan You, Liyang Sai

**Affiliations:** ^1^School of Psychology, Shenzhen University, Shenzhen, China; ^2^Shenzhen Key Laboratory of Affective and Social Cognitive Science, Shenzhen, China; ^3^Department of Psychology, Hangzhou Normal University, Hangzhou, China

**Keywords:** social emotion, pleasant emotions, emotional processing, subcultural differences, event-related potentials

## Abstract

While previous studies have shown that East-West cultural differences exist in processing different emotional stimuli, potential subcultural differences within a large cultural group are not well understood. In this study, we examined subcultural differences in the event-related potential (ERP) modulations in the brain, during processing social positive and non-social positive stimuli among 21 Han Chinese and 21 Uygur Chinese. Results showed that the magnitudes of P2, N2, and late positive potential (LPP) were larger in Uygur Chinese than in Han Chinese. For social positive stimuli, the P2 and LPP 300–600 were larger in Uygur Chinese than in Han Chinese. However, there was no significant difference in non-social positive stimuli. These results indicated that social positive emotions were more prioritized in emotional processing among Uygur Chinese than Han Chinese. These findings contribute to the growing literature on subcultural differences in processing different types of positive stimuli.

## Neurophysiological Evidences of Subcultural Differences in Processing Social and Non-Social Positive Emotions

Even though emotion has biological underpinnings, it is significantly influenced by cultural differences (Murata et al., 2013). Recent research on cultural neuroscience indicates that different cultures create a chance for non-shared biology by impacting the development of individuals’ neural system (Gutchess and Goh, 2013). Prior research on neuroplasticity suggests that norm-guided regulation can give rise to changes in the psychobiological structures and processes of individuals (Kitayama and Park, 2010, for review).

Several cross-cultural studies have shown that Chinese have different beliefs, attitudes, and regulation tendencies toward different emotions as compared to their Western counterparts, especially for positive emotions (Miyamoto and Ma, 2011; Deng et al., 2019). This reflects the distinctive cultural values regarding emotions in Chinese culture (Deng et al., 2018, 2019; Ma et al., 2018). Therefore, this exploratory study aims to test whether culture shapes electro-cortical responses during processing of positive emotions by using an event-related potential (ERP) measure.

## Culture and Positive Emotions

Social norms surrounding emotional responses are different across cultures (Ishii, 2013). People are encouraged to act in accordance with their social norms. For example, people have been observed to regulate, express, and even experience emotions in a manner that is culturally appropriate (Miyamoto et al., 2014). Cultural differences in emotional beliefs and regulation have been observed to be the greatest in positive emotions that reflect the different cultural scripts between Chinese and their Western counterparts (Joshanloo et al., 2013).

An important and distinctive script of Chinese culture is dialectical thinking. It is defined as a cognitive style and habituate behavioral tendency that believing reality is constantly changing and a tolerance of contradictions (Spencer-Rodgers et al., 2009). First, due to engaging in dialectical thinking, Chinese emphasize the potential negative consequences of positive emotions (Deng et al., 2016). Most of the prior research on emotions in the context of Western culture makes the assumption that happiness is desirable and worth pursuing. However, Chinese are more likely to perceive negative aspects of happiness and prefer to feel less happy in their daily lives (Deng et al., 2018, 2019). Further, Chinese are less likely than European Americans to savor positive emotions (Miyamoto and Ma, 2011). Chinese people prefer to decrease their pleasant emotions in order to avoid the potential negative consequences of pleasant emotions (Wei et al., 2013).

Second, contradiction states are in tandem with the dialectical thinking style of Chinese (Ma et al., 2018). The principle of change is emphasized in some Chinese philosophical traditions, such as Taoism. Happiness is constantly evolving and in the state of flux. Pleasant and unpleasant emotional states could change. This implies that pleasant emotions might be co-occurring with unpleasant emotions (Spencer-Rodgers et al., 2009).

In brief, the existing empirical evidence indicates that processing of positive emotions reflect distinctive cultural values regarding emotions in Chinese culture. Therefore, it is important for researchers to explore positive emotion processing in Chinese culture.

## Subcultural Differences Between Han Chinese and Uygur Chinese

In previous cultural studies, the most common division is comparison between Eastern and Western individuals, or specific nations (e.g., China vs. United States). However, recent studies highlight the importance for researchers to focus on local subcultures when comparing vastly diverse cultural identities (Gutchess and Goh, 2013). For example, Deng et al. (2016) suggested that there were subcultural differences in the attitudes toward happiness and mixed emotional experiences within Chinese culture. Compared to Mongolian Chinese, Han Chinese showed stronger associations between implicit contra-hedonic attitudes toward happiness and mixed emotions during pleasant emotional events. Although subcultural differences were emphasized in the recent studies, they are still poorly understood.

Being a multi-ethnic country, China is comprised of diverse cultural systems. For example, the mainstream Chinese culture is marked by Han Chinese culture, which is shaped by Confucian (Sundararajan, 2015). The experience and regulation of positive emotions are dialectical and are influenced by contra-hedonism (Deng et al., 2016). Like Han Chinese, Uygur Chinese also value interdependence and collectivism. Also, a previous study suggested that Uygur Chinese had lower level of constraint and higher hedonism than Han Chinese (Lv and Wang, 2012). Although there is no contradiction between ethnic and state identities, Uygur Chinese are emotionally more attached to Xinjiang region and manifest strong ethnic and local identity (Yee, 2005). Territorial autonomy of their residence makes Uygur Chinese less susceptible to mainstream Han culture.

The Chinese population has a larger proportion of Han Chinese than Uygur Chinese. Han culture represents mainstream cultural values of Chinese. As the dominant culture, the history and tradition of Han Chinese was well preserved until now. The impact of Confucian is still embedded in the minds of Han Chinese and continues to influence their daily emotional lives (Sundararajan, 2015).

Although both ethnic groups value collectivism, Uygur Chinese are found to have better knowledge of the social self than Han Chinese (Hu, 2000). Uygur Chinese value group integration and unity. Prior research found that Han people tended to emphasize the relational self, whereas Uyghur people tended to emphasize the collective self (Mamat et al., 2014). In a prior study, Uygur Chinese reported lower emotion regulation than Han Chinese (Lv and Wang, 2012). Moreover, Uygur Chinese reported higher level of positive as well as negative emotional experiences than Han Chinese in their daily lives (Lv and Wang, 2012). However, such findings from previous research are mostly based on behavioral measures. Little is known about the psychophysiological processes underlying the processing of positive emotions. Thus, the present study aimed to explore subcultural differences in the emotional processing of social positive and non-social positive stimuli using ERP measures.

## Psychophysiological Markers for Emotional Processing

Examining cultural differences in the biomarkers for emotional processing has potential to provide an important supplement to behavioral measures (An et al., 2018). As an early component, P2 is the prominent frontal component occurring approximately 200 ms prior N2 (Lamm and Lewis, 2010). It indicates arousal and emotional reaction to emotional stimulus. P2 is modulated by inter-modal attention effects as it is sensitive to stimulus discrimination (De Tommaso et al., 2008). For example, compared with neutral facial expression, emotional facial expression (e.g., painful) increased Chinese participants’ frontal/central P2. This effect was only found for Asian faces but not for Caucasian faces (Sheng and Han, 2012).

N2, another important early component relevant to emotional processing, is the largest negative deflection with a medial-frontocentral topography between 200 and 300 ms following stimulus onset (Ladouceur et al., 2010). N2 has been observed to be sensitive to the processing of emotional information. For example, previous research shows that emotion-related changes in N2 activation occurred within the tasks that were designed to induce negative emotions (e.g., go no-go task). Further, Lewis et al. (2007) found larger N2 amplitude for negative stimuli than positive stimuli (e.g., angry vs. happy faces). The N2 component is also used to index cultural differences. For example, a previous study found larger N2 for European Americans than for Asian Americans during a low-frequency target detection task (Kitayama and Murata, 2013).

As a late component, late positive potential (LPP) is a typically sensitive biomarker of emotional processing. LPP is long-lasting positivity at the midline-parietal electrode (e.g., Pz) which peaks around 300–400 ms after stimulus onset (Murata et al., 2013). It reflects attention given to emotional stimuli and the recruitment of prefrontal cortical resources associated with effective cognitive control (Dennis and Hajcak, 2009). For example, enhanced LPP in response to angry versus neutral faces was found in 8- to 14-year-old children (Wessing et al., 2015). LPP can be modulated by reappraisal with decreased amplitudes during emotion regulation (Langeslag and Van Strien, 2010). In another cross-cultural study, Easterners showed a significant decrease of the parietal LPP in the condition of expression suppression. However, no attenuation of the parietal LPP was found when European Americans tried to suppress their emotional expressions (Murata et al., 2013).

## The Present Study

Based on the existing literature, Chinese culture displays distinctive cultural values on emotions in the processing of positive emotions. As an addition to previous research (Miyamoto and Ma, 2011; Deng et al., 2018, 2019), we focused on positive emotions and highlighted the importance of positive emotions in the context of Chinese culture. To date, virtually most studies about emotional processing made cross-cultural comparisons between East Asians and Westerners. In the present study, further emphasis was placed on local subcultures and subcultural differences within a diverse cultural background. However, there was little empirical evidence for the subcultural differences in emotional processing between Han and Uygur Chinese. It is impossible to rule out all the possible reasons for the differences in the processing of positive emotions. Therefore, it would be more convincing and feasible for the present study to adopt the “just minimal difference” approach (Cohen, 2007; Uskul et al., 2008), which allows keeping constant as many potentially confounding variables as possible while exploring the subcultural difference of interest (in this case, different ethnicities). To the authors’ knowledge, subcultural differences in emotional processing have not been examined using ERP yet. In the present study, ERP was used as an objective measure to complement behavioral measures.

The goal of the present study was to explore and examine potential subcultural differences in the electro-cortical responses during emotional processing of social and non-social positive emotions. As the typical biomarkers of emotional processing, subcultural differences in the early ERP components (e.g., P2 and N2) and later ERP components (e.g., LPP) were examined. Uygur Chinese is an important ethnic group, and Han Chinese is a majority ethnic group in China. Thus, subcultural differences between these two groups were explored. Since previous research suggests that Uygur Chinese experience higher level of positive and negative emotions than Han Chinese in their daily lives (Lv and Wang, 2012), the authors predicted that Uygur Chinese would show larger magnitudes of ERP components (e.g., P2, N2, and LPP) during emotional processing. Compared to the culture of Han Chinese, Uygur culture views people as more interconnected in a specific social context (Tang, 2008). They emphasize group integration and have better knowledge of social self than Han Chinese (Mamat et al., 2014). Thus, subcultural differences in the processing of social and non-social emotions were expected in this exploratory study.

## Materials and Methods

### Participants

The present study adopted the “just minimal difference” approach (Cohen, 2007; Uskul et al., 2008), which allowed keeping constant as many potentially confounding variables as possible. To focus on our research interest (in this case, different ethnicities), all of the participants were university students. As the university students, the two subcultural groups fell in the same economic social status and similar level of cognitive ability. Twenty one Han Chinese (9 male, 12 female, *M*_age_ = 20.33, SD = 1.08) and 21 Uygur Chinese (10 male, 11 female, *M*_age_ = 20.95, SD = 0.95) undergraduates from Shenzhen University participated in the study in exchange for 50 RMB (equivalent to 8 dollar). This cell size was chosen to exceed the recommendations for minimum sample size (i.e., 20 participants per condition; see Simmons et al., 2011). All Han Chinese participants self-identified as Han Chinese. Although Shenzhen is a migrant city, all Han Chinese participants were Shenzhen local citizens and Cantonese. Previous study indicated that Cantonese regions and people in the Cantonese regions have retained the most traditional models of the Han Chinese culture (Huang, 1998; Xu and Xu, 2011; Zeng, 2016). Therefore, participants in the present study were a representative sample of enculturated Han Chinese. All Uygur Chinese participants were born in and have lived in Xinjiang Uygur Autonomous Region of China before attending university. They self-identified as Uygur Chinese. They have lived in Shenzhen for 1–4 years. Pearson’s correlation analysis (two-tailed) was conducted between the duration of stay of Uygur participants in Shenzhen and the amplitude of ERPs. No correlations were found to be significant (all *p*’s >0.05). Therefore, the duration of stay of the participants in Shenzhen was not correlated with the studied ERPs. All participants were right-handed and had normal or corrected-to-normal vision. Written informed consent was obtained from the participants before the study. The research protocol was approved by the Institutional Reviewing Board at Shenzhen University.

### Stimulus Materials

Thirty social positive pictures (valence: *M* = 6.99, SD = 0.33; arousal: *M* = 5.97, SD = 0.81), 30 non-social positive pictures (valence: *M* = 7.16, SD = 0.48; arousal: *M* = 5.66, SD = 0.34), and 30 neutral pictures (valence: *M* = 5.71, SD = 0.93; arousal: *M* = 5.72, SD = 0.54) were taken from the Chinese Affective Picture System (CAPS; Bai et al., 2005) and the International Affective Picture System (IAPS; Lang et al., 2005) based on the CAPS and IPAS normative ratings. The neutral pictures had depictions of neutral objects, such as household objects. The non-social positive picture set included pictures of pleasant-looking animals and appetizing food. The social positive picture set had pictures of pleasant social situations. Social situations were defined as situations where people interacted with each other (Silvers et al., 2012). Results from pre-tests confirmed that social pictures reminded participants of social situations more than non-social stimuli (*p* < 0.001); however, they did not differ in terms of valence (*p* > 0.05) and arousal (*p* > 0.05). Both valence and arousal were rated on a 9-point scale, with higher ratings for valence corresponding to more pleasant feelings and higher ratings for arousal corresponding to more arousing feelings. Each picture was displayed in color and occupied the entirety of a 19-inch monitor. The task was administered using the E-Prime software.

### Procedures and Measures

An affective picture processing task was used to assess participants’ neural responses to social positive, non-social positive, and neutral stimuli. The affective picture processing task was the most classic paradigm in the area of emotional processing and has been widely used in different culture samples (Hajcak and Dennis, 2009; Gao et al., 2010; Deng et al., 2018). After giving a brief description of the experiment, electroencephalograph (EEG) sensors were attached to the participant followed by detailed instructions for the task. Participants first viewed a practice series of nine pictures displayed on the screen. After confirming that they understood the experiment procedure, the test experiment was begun. There were 90 pictures in the test experiment. Each of the 90 pictures was randomly displayed twice, with a total of 180 trials. At the beginning of each trial, a fixation was presented for 500 ms. Next, a stimulus picture was presented for 2,000 ms. The continuous EEG was recorded during the stimuli presented. Next, all participants were asked to rate each picture on the valence and arousal scales of the self-assessment manikin (Lang et al., 2005) ranging from 1 to 5. For the valence rating, participants were told to rate each picture on this scale based on how happy the picture made them feel. For the arousal rating, participants were told to rate the pictures based on the strength of their feeling in response to the picture.

### Psychophysiological Recording, Data Reduction, and Analysis

The continuous EEG was recorded with a 64-channel amplifier (BrainAmp, Brain Products, Germany) based on the 10/10 system, with two electrodes placed on the left and right mastoids. The EEG was sampled at 500 Hz. Impedance was kept below 5 kΩ. The data were re-referenced offline to the averaged mastoid references and band pass filtered from 0.1–30 Hz. Eye movements and blink artifacts were corrected by using the independent component analysis (ICA) algorithm implemented in Brain Vision Analyzer 2.0 (“Brain Products,” Germany). Data were segmented in epochs from 200 ms before the onset of stimuli until 1,500 ms after the onset. ERPs were constructed by separately averaging the three picture types (social positive, neutral, and non-social positive). For each ERP average, the average activity in the 200 ms picture onset served as the baseline. Trials with artifacts exceeding ±80 μV were excluded from further analysis. The mean number of valid epochs averaged per condition was 55.48 (92.04%) for non-social positive stimuli, 55.57 (92.62%) for social positive stimuli, and 55.24 (92.06%) for neutral stimuli in Han Chinese. While the mean number of valid epochs averaged per condition was 48.05 (80.08%) for non-social positive stimuli, 45.38 (5.63%) for social positive stimuli, and 47.29 (78.81%) for neutral stimuli in Uygur Chinese.

In previous studies, P2 was the prominent frontal component occurring around 200 ms prior to N2 (Lamm and Lewis, 2010). Based on the existing literature and visual inspection, in the present study, P2 was evaluated at Fz as the average activity between 130 and 200 ms following stimulus onset. The N2 was evaluated at Cz as the average activity between 210 and 260 ms following stimulus onset (De Tommaso et al., 2008). The LPP was defined as the average activity at Pz (Langeslag and Van Strien, 2010; Murata et al., 2013) in three time windows following stimulus onset: 300–600 ms (early window), 600–1,000 ms (middle window), and 1,000–1,500 ms (late window). The valence and arousal ratings, P2, N2, and LPP were statistically evaluated using SPSS 20.0. Greenhouse-Geisser correction was applied to *p*’s associated with multiple-df comparisons.

## Results

### Valence and Arousal Rating Data

For the valence ratings, there was a significant main effect for stimuli types, *F*(2,80) = 50.52, *p* < 0.001, $\eta_{p}^{2}$ = 0.56. Valence ratings of social positive stimuli were higher than those for non-social positive stimuli (*p* = 0.004) and neutral stimuli (*p* < 0.001). There was no significant main effect for ethnic group, *F*(1,40) = 0.13, *p* = 0.722, $\eta_{p}^{2}$ = 0.003. Further, interaction between stimuli types and ethnic group was not found to be significant, *F*(2,80) = 2.26, *p* = 0.127, $\eta_{p}^{2}$ = 0.053 (Table 1).

**Table 1:** Means and standard deviations for subjective valence and arousal rating between Han Chinese (*n* = 21) and Uygur Chinese (*n* = 21) in non-social positive, social positive, and neutral conditions.

	Han Chinese				Uygur Chinese			
Stimulus type	Valence		Arousal		Valence		Arousal	
	*M*	SD	*M*	SD	*M*	SD	*M*	SD
Social positive	3.59	0.89	3.11	0.83	3.63	0.80	3.01	0.89
Non-social positive	3.38	0.47	3.07	0.64	3.22	0.76	2.85	0.86
Neutral	2.40	0.44	2.72	0.64	2.70	0.73	2.54	0.66

For the arousal rating, there was a significant main effect for stimuli types, *F*(2,80) = 12.38, *p* < 0.001, $\eta_{p}^{2}$ = 0.236. Arousal ratings of social positive stimuli were higher than neutral stimuli (*p* < 0.001). Arousal ratings of non-social positive stimuli were higher than neutral stimuli (*p* = 0.001). There was no significant difference between social positive and non-social positive stimuli (*p* > 0.05). There was no significant main effect for ethnic group, *F*(1,40) = 0.628, *p* = 0.433, $\eta_{p}^{2}$ = 0.015. Further, interaction between stimuli types and ethnic group was not significant, *F*(2,80) = 0.218, *p* = 0.77, $\eta_{p}^{2}$ = 0.005.

### P2 Component

**Figure 1:**
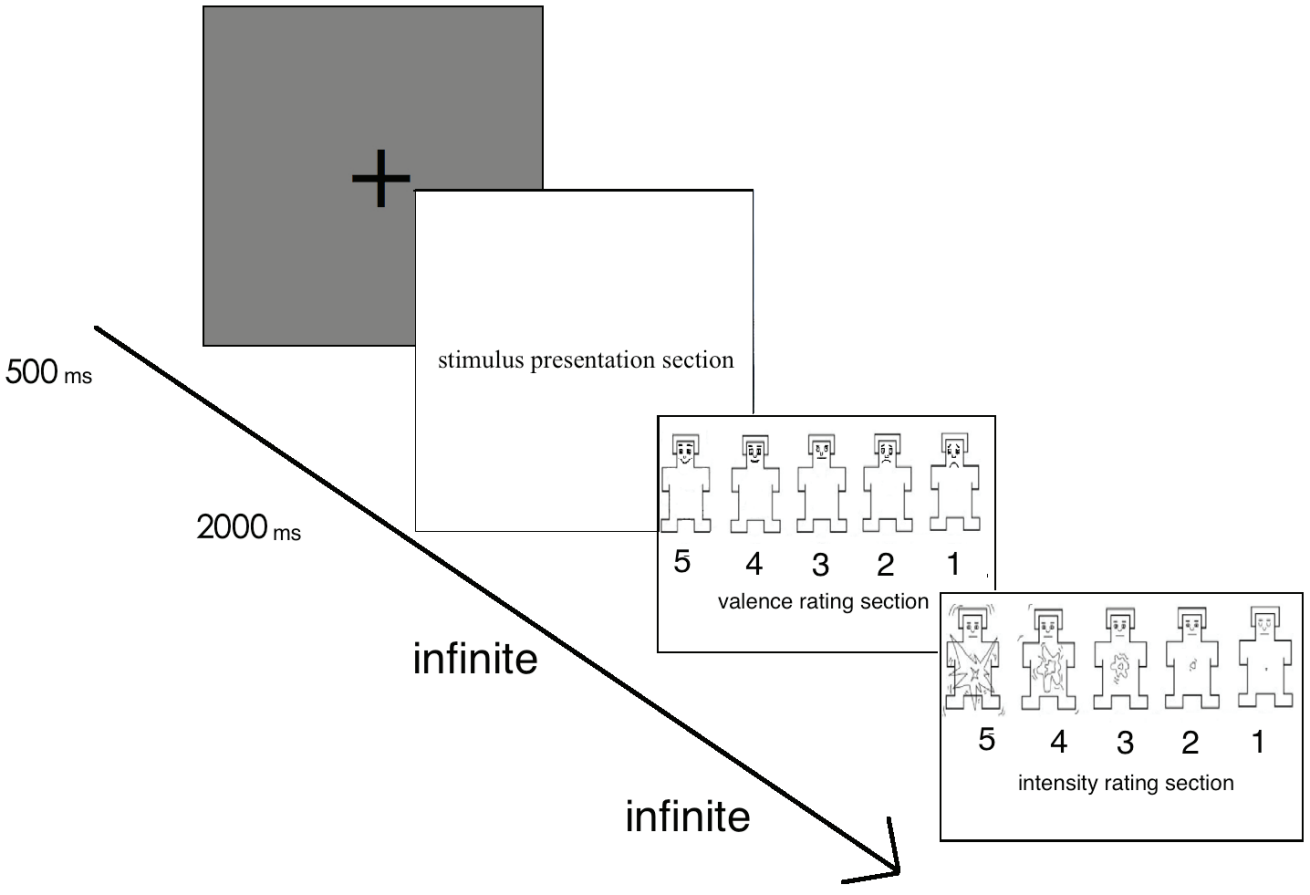
Procedure of each trial.

**Figure 2:**
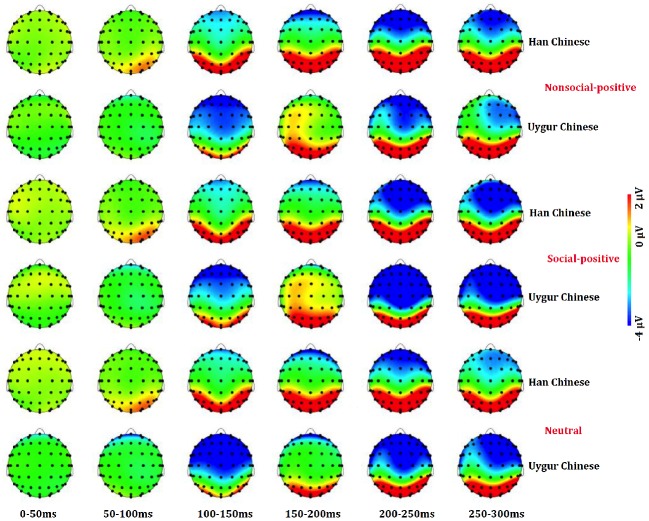
Scalp distributions in mean activity to non-social positive (**Top**), social positive (**Middle**), and neutral stimuli (**Bottom**) at 0–300 ms between Han Chinese (*n* = 21) and Uygur Chinese (*n* = 21).

**Figure 3:**
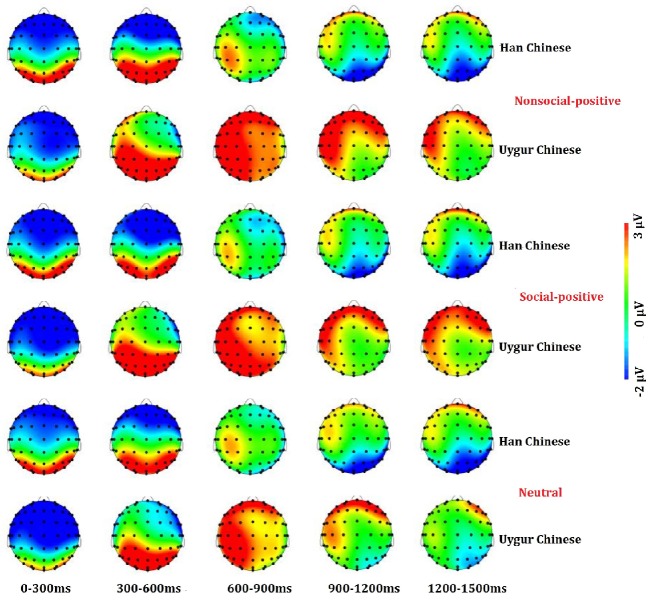
Scalp distributions in mean activity to non-social positive (**Top**), social positive (**Middle**), and neutral stimuli (**Bottom**) at 0–1,500 ms between Han Chinese (*n* = 21) and Uygur Chinese (*n* = 21).

**Figure 4:**
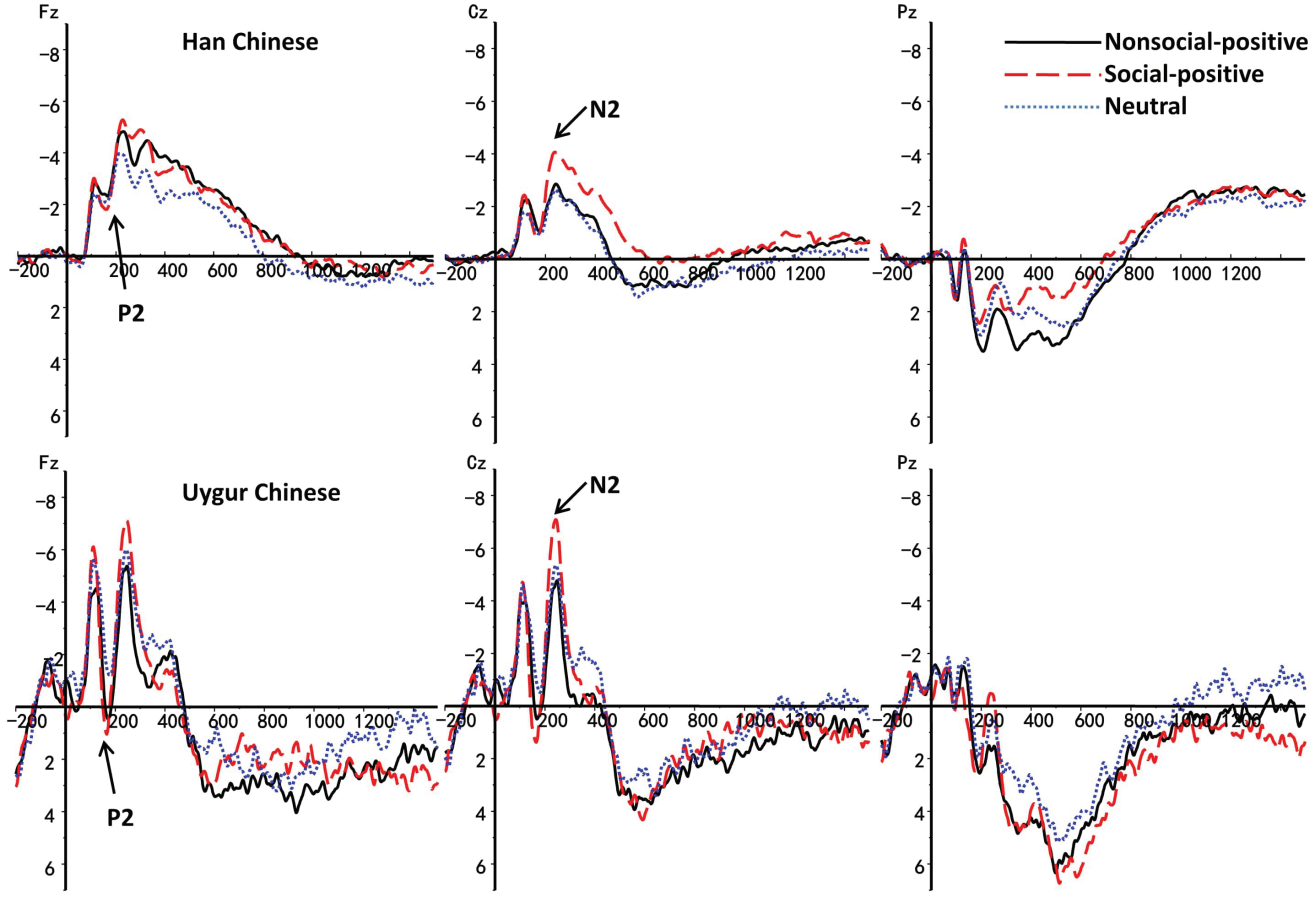
Stimulus-locked ERPs elicited by non-social positive, social positive, and neutral stimuli between Han Chinese (*n* = 21) and Uygur Chinese (*n* = 21) at Fz, Cz, Pz recording sites. Stimulus onset occurred at 0 ms. Activation is shown as negative down.

The main findings with regard to the P2 component are illustrated in Figures 1–4. A 2 (ethnic group: Han Chinese vs. Uygur Chinese) × 3 (stimulus type: social positive vs. non-social positive vs. neutral) repeated measures ANOVA was conducted to examine the subcultural differences in P2 component when viewing different emotional stimuli. Results showed a significant main effect for stimuli types, *F*(2,80) = 9.39, *p* < 0.001, $\eta_{p}^{2}$ = 0.19. There was a significant main effect for ethnic group, *F*(1,40) = 4.839, *p* = 0.34, $\eta_{p}^{2}$ = 0.108. P2 was larger in Uygur Chinese than in Han Chinese, *p* < 0.05. Interaction between stimuli types and ethnic group was significant, *F*(2,80) = 4.03, *p* = 0.022, $\eta_{p}^{2}$ = 0.092. For social positive stimuli, P2 was larger in Uygur Chinese than in Han Chinese (*p* = 0.007). For non-social positive stimuli and neutral stimuli, there was no significant difference between Uygur Chinese and Han Chinese (both *p* > 0.05).

### N2 Component

The main findings with regard to the N2 component are illustrated in Figures 1–4. A 2 (ethnic group: Han Chinese vs. Uygur Chinese) × 3 (stimulus type: social positive vs. non-social positive vs. neutral) repeated measures ANOVA was conducted to examine the subcultural differences in N2 component when viewing different emotional stimuli. Results showed a significant main effect for stimuli types, *F*(2,80) = 14.73, *p* < 0.001, $\eta_{p}^{2}$ = 0.269. N2 was larger for social positive stimuli as compared to non-social positive stimuli (*p* = 0.001). N2 was larger for non-social positive stimuli than for neutral stimuli, *p* < 0.001. There was a significant main effect for ethnic group, *F*(1,40) = 4.31, *p* = 0.044, $\eta_{p}^{2}$ = 0.097. N2 was larger in Uygur Chinese than in Han Chinese, *p* < 0.05. Interaction between stimuli types and ethnic group was not significant, *F*(2,80) = 0.27, *p* = 0.738, $\eta_{p}^{2}$ = 0.007.

### Late Positive Potential 300–600 (Early Window 300–600 ms)

**Figure 5:**
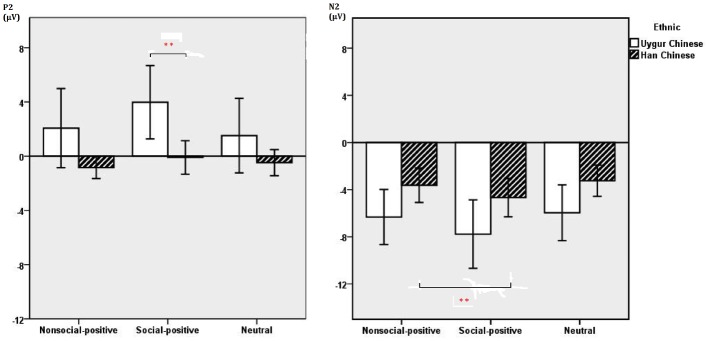
Average P2 (**Left**), N2 (**Right**) amplitudes of non-social positive, social positive, and neutral stimuli at Fz and Cz recording sites between Han Chinese (*n* = 21) and Uygur Chinese (*n* = 21). Error bars represent ±2 standard errors. **p < 0.05.

**Figure 6:**
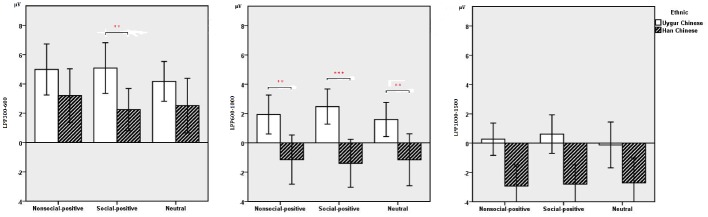
Average LPP amplitudes of non-social positive, social positive, and neutral stimuli in the time window of the early LPP (300–600 ms; **Left**), middle LPP (600–1,000 ms; **Middle**) and late LPP (1,000–1,500 ms; **Right**) between Han Chinese (*n* = 21) and Uygur Chinese (*n* = 21). Error bars represent ±2 standard errors. **p < 0.05, ***p < 0.001.

The main findings with regard to the LPP components are illustrated in Figures 3–6. 2 (ethnic group: Han Chinese vs. Uygur Chinese) × 3 (stimulus type: social positive vs. non-social positive vs. neutral) repeated measures ANOVA was conducted to examine the subcultural differences in LPP components when viewing different emotional stimuli.

For the LPP 300–600, results showed no significant main effect for stimuli types, *F*(2,80) = 2.399, *p* = 0.103, $\eta_{p}^{2}$ = 0.057. There was a significant main effect for ethnic group, *F*(1,40) = 6.142, *p* = 0.018, $\eta_{p}^{2}$ = 0.133. LPP 300–600 was larger in Uygur Chinese than in Han Chinese, *p* < 0.05. Interaction between stimuli types and ethnic group was significant, *F*(2,80) = 6.142, *p* = 0.004, $\eta_{p}^{2}$ = 0.133. For social positive stimuli, LPP 300–600 was larger in Uygur Chinese than in Han Chinese, *p* = 0.002. For non-social positive stimuli and neutral stimuli, there was no significant difference between Uygur Chinese and Han Chinese (both *p*’s > 0.05). For Uygur Chinese, there were significant differences in the LPP 300–600 in different emotional conditions (social positive stimuli > neutral stimuli, *p* = 0.019). For Han Chinese, there were significant differences in the LPP 300–600 in different emotional conditions (non-social positive stimuli > social positive stimuli, *p* = 0.017).

### Late Positive Potential 600–1,000 (Middle Window 600–1,000 ms)

For the LPP 600–1,000, results showed no significant main effect for stimuli types, *F*(2,80) = 0.233, *p* = 0.756, $\eta_{p}^{2}$ = 0.006. There was a significant main effect for ethnic group, *F*(1,40) = 16.673, *p* < 0.001, $\eta_{p}^{2}$ = 0.294. LPP 600–1,000 was larger in Uygur Chinese than in Han Chinese, *p* < 0.001. Interaction between stimuli types and ethnic group was marginally significant, *F*(2,80) = 1.41, *p* = 0.053, $\eta_{p}^{2}$ = 0.075.

### Late Positive Potential 1,000–1,500 (Late Window 1,000–1,500 ms)

For LPP 1,000–1,500, results showed no significant main effect for stimuli types, *F*(2,80) = 0.183, *p* = 0.813, $\eta_{p}^{2}$ = 0.005. There was a significant main effect for ethnic group, *F*(1,40) = 13.116, *p* = 0.001, $\eta_{p}^{2}$ = 0.247. LPP 1,000–1,500 was larger in Uygur Chinese than in Han Chinese, *p* = 0.001. Interaction between stimuli types and ethnic group was not significant, *F*(2,80) = 1.724, *p* = 0.188, $\eta_{p}^{2}$ = 0.041.

## Discussion

Culture not only affects individual behaviors but also neural processes associated with affective processing, such as emotional perception and emotional processing (Matsumoto and Hwang, 2012). This exploratory study tried to examine the subcultural differences in the positive emotional processing by using a neuroscience technique. We found subcultural differences in the electro-cortical responses during emotional processing of social and non-social positive emotions. Drawing on previous research on positive emotions in Chinese cultural background, the authors hypothesized that Uygur Chinese and Han Chinese would differ in the early and later ERP components (e.g., P2, N2, and LPP) during emotional processing. Specifically, compared to Han Chinese, we expected that Uygur Chinese would prioritize social positive emotions.

Consistent with our hypotheses, there were significant main effects for ethnic group in all the ERP components. The amplitudes of P2, N2, and LPP were larger in Uygur Chinese than in Han Chinese. This is in line with previous research, which has found significantly larger amplitudes for European Americans than for Asian Americans in early ERP components (e.g., N2, Kitayama and Murata, 2013). N2 is the negativity at the frontal region of the brain typically observed around 200 ms post stimulus. It has been observed to reflect a brain response signaling the discrimination of a target and orienting attention. P2 is an early ERP component that reflects enhanced attention given to biologically important stimulus (De Tommaso et al., 2008). If replicated, subcultural differences in P2 and N2 in the present study might imply cultural difference was deep-rooted in the early stage of perceptual and attentional processing.

Culture can affect stimulus-oriented attention in early stages. As stated, Uygur Chinese experienced higher level of positive and negative emotions than Han Chinese in their daily lives (Lv and Wang, 2012). Previous behavioral studies as well as the present ERP study found that Han Chinese showed a lower level of cortical activation and experience of emotional stimuli as compared to other cultural groups (Lim, 2016). Higher level of early stimulus-oriented attention can affect Uygur Chinese’s emotional experience in the later stage of emotional processing. Findings from this study about the subcultural differences in the early ERP components (indicative of early orientation and attention) could serve as a foundation for future studies examining other components of emotional responding and their cultural predictors.

Late positive potential (LPP) is an attention-sensitive positive slow wave which has shown to be responsive to emotion-evoking stimulus (Langeslag and Van Strien, 2010). It is a typical and important biomarker of emotion regulation and is involved in the recruitment of prefrontal cortical resources associated with cognitive control (Dennis and Hajcak, 2009). Similar to the findings for early ERP components, the amplitudes of LPP were larger in Uygur Chinese than in Han Chinese in all time windows. In contrast to previous cross-cultural studies, Han Chinese showed a lower electro-cortical activation in response to emotional stimuli than their western counterparts as well as Uygur Chinese. This suggests that Han Chinese could have lower attentional processing and reactivity to emotional stimuli and they recruit less cortical resources during later stages of emotional processing.

While examining the processing of different types of positive stimuli, we found interactions between stimulus types and ethnic groups. For social positive stimuli, P2 and LPP 300–600 were larger in Uygur Chinese than in Han Chinese. However, there was no significant difference in other types of stimuli. This indicates that the subcultural differences in emotional processing between Uygur Chinese and Han Chinese are specific to the processing of social positive stimuli. P2 and LPP are known to increase as a function of attentional and emotional significance. Although we did not directly measure any culture-related factors, the subcultural differences in emotion regulation between Uygur Chinese and Han Chinese could be possible explanations for the subcultural differences in emotional processing between groups. In contrast to Uygur Chinese, the importance of relational self was more salient than the collective self in Han Chinese because of the influence by the Confucian (Mamat et al., 2014). Different from Uygur Chinese, creating “Guanxi (关系)” is essential to achieve success for Han Chinese (Sundararajan, 2015). Guanxi is defined as the functional relational ties in personalized social networks of power. In Han Chinese culture, self-constraint is emphasized. People are discouraged from expressing happiness in front of others. Thus, the control of social positive emotions might be a possible reason for the low activations of Han Chinese during emotional processing.

Another possible explanation for the subcultural differences in emotional processing between groups could be that Han Chinese group is part of the dominant Confucian culture which advocates moderation (Sundararajan, 2015). The culture of moderation of the self emphasizes the pursuit of the “*middle way*.” For example, the ideal emotional state for Han Chinese is usually calm and peaceful but not happy. The famous Chinese poem stated: “Neither pleased by the material things, nor saddened by one’s personal losses (不以物喜, 不以己悲).” Their contra-hedonic emotional attitudes may dispose the Han Chinese to engage less with their emotions as well as to express them less (Deng et al., 2016). In this sense, the contra-hedonic emotional attitudes might be a possible reason for the low activation of Han Chinese during emotional processing. However, it is worth noting that the current study was the first to explore the subcultural differences in the psychophysiological base of emotional reactivity. It did not examine any culture-related data that accounted for the subcultural differences in emotional processing. Further examinations for the potential mechanism for the subcultural differences in emotional processing in future study are needed.

In the present study, we did not find any significant main effect between groups based on our valence and arousal rating data. Because of the examining a different component of emotional reactivity, ERP is a more sensitive technique to examine psychological processing than self-report. Therefore, discrepancy between valence and arousal rating data and ERP data are not unusual. Different from the valence and arousal rating results, subcultural differences in the ERP data were robust across time windows. Not only the early ERP components (P2 and N2) that represented bottom-up attentional processes (e.g., N2, salience detection, stimulus driven orienting, and mind wandering) but also the later ERP components (LPP) that represented top-down regulatory processes (e.g., LPP, attention control, and emotion regulation) were significantly different between Uygur and Han Chinese. Especially for LPP, it reflects the recruitment of prefrontal cortical resources associated with cognitive control (Dennis and Hajcak, 2009). Uygur Chinese was significantly different with Han Chinese in responses to different types of positive stimuli across all time windows. It might imply that subcultural differences were not only due to the early cognitive mechanism but also the higher levels of conscious evaluation and other social factors.

Also, as shown in Table 2 and Figure 6, we found that there were significant differences in ERPs in the neutral condition. In the traditional research of emotional processing, neutral stimuli are used as the control conditions or baseline comparisons with the emotional stimuli (Hajcak and Dennis, 2009; Langeslag and Van Strien, 2010). They are neutral in the content and property (e.g., household objects), but they are not non-emotional. The pleasant ratings (valence of the stimuli) of the neutral stimuli were normally in a moderate level. Therefore, the ERP differences in the neutral conditions in our study might indicate the differences in the baseline of the emotional processing between subcultural groups.

**Table 2:** Means, standard deviations, and the testing parameters of the group differences between Han Chinese (*n* = 21) and Uygur Chinese (*n* = 21) in the Studied ERPs (P2, N2, Early LPP, Middle LPP, and Late LPP) in non-social positive, social positive, and neutral conditions.

			Han Chinese		Uygur Chinese		*t*	df	*p*	*D*	95% CI	
P2		Non-social positive	−8.43	1.77	2.07	6.42	2.004	23.042	0.057	0.835	−0.094	5.92
		Social positive	−0.09	2.72	3.98	5.93	2.859	28.059	0.008	1.079	1.15	6.99
		Neutral	−0.47	2.12	1.52	6.03	1.426	24.843	0.166	0.572	−0.88	4.86
N2		Non-social positive	−3.63	3.19	−6.32	5.13	−2.039	33.493	0.049	−0.705	−5.37	−0.01
		Social positive	−4.68	3.58	−7.77	6.37	−1.943	31.459	0.061	−0.693	−6.35	0.15
		Neutral	−3.25	2.92	−5.96	5.20	−2.058	31.463	0.045	−0.734	−5.36	−0.06
LPP	300–600	Non-social positive	3.03	3.18	4.91	3.83	1.737	40	0.09	0.549	−0.31	4.08
		Social positive	1.62	3.26	5.38	4.21	3.234	40	0.002	1.023	1.41	6.11
		Neutral	2.34	3.29	4.33	3.39	1.958	40	0.057	0.619	−0.06	4.04
	600–1,000	Non-social positive	−0.11	3.06	2.84	2.70	3.301	40	0.002	1.044	1.14	4.74
		Social positive	−0.60	3.09	3.67	2.79	4.710	40	0.000	1.489	2.44	6.11
		Neutral	−0.03	3.00	2.67	2.76	3.041	40	0.004	0.962	0.91	4.51
	1,000–1,500	Non-social positive	−2.40	2.64	0.41	2. 29	3.682	40	0.001	1.164	1.27	4.35
		Social positive	−2.57	2.81	1.01	3.17	3.869	40	0.000	1.223	1.71	5.45
		Neutral	−2.01	3.08	0.24	3.32	2.279	40	0.028	0.721	0.25	4.25

To focus on our primary contrast of interest, this study adopted a “just minimal difference” approach to keep constant as many potentially confounding variables as possible (Cohen, 2007; Uskul et al., 2008). Even so, the duration of stay of Uygur participants in Shenzhen was controlled during the data analysis. They were all university students. All of the Uygur participants had lived in Xinjiang province before entering university. This feature of the sample allowed us to minimize any potential effects of the tendency of participants to self-select to live in a chosen ecoculture and acculturation to a different ecoculture.

## Limitation and Future Directions

The present study focused on the electro-cortical responses to two types of positive stimuli during emotional processing between two sub-groups of people living in Chinese cultural context. However, it is unclear whether there is a difference in the way different ethnic groups in China perceive and regulate their pleasant emotions in real life. After all, people are not expected to report and rate their emotional valance and arousal level just like what they did in the experiment when they felt happy in a real emotional setting. This can be an intriguing direction for further studies. Furthermore, the current literature on cultural differences between Chinese and their western counterparts suggests that cultural values lead Han Chinese to have contra-hedonic attitudes toward happiness (Deng et al., 2016). However, the way these attitudes toward emotions impact emotional processing needs to be examined. Although the duration of stay of Uygur participants in Shenzhen was not correlated with the studied ERPs, Uygur participants could have been influenced by the local culture. Therefore, it is not clear if they truly represent the subculture of Xinzhang.

For Han Chinese, we did not examine how Confucian philosophy influences their attitude toward emotion or emotional processing. Whether subcultural differences remain among Han Chinese living in Xinjiang and Han Chinese who are less influenced by the Confucian cultural values are still unknown. Further research is required to include the assessment of native Uygur Chinese, Han Chinese living in Xinjiang, and Han Chinese who are less influenced by the Confucian cultural values.

Besides the potential eco-cultural differences between Uygur and Han Chinese, there may be some other variables which may also influence the sub-cultural differences, such as different cognitive styles. In the present study, some early ERP components (P2 and N2) showed significant differences between groups. Given that the P2 and N2 represented bottom-up attentional processes, different cognitive styles might be a possible reason for the subcultural differences in the positive emotional processing between Uygur and Han Chinese. However, to our knowledge, there is little research examining subcultural differences in the basic cognitive mechanisms between Uygur and Han Chinese. To our knowledge, we were the first to conduct an exploratory study to examine the subcultural differences in the positive emotional processing between Uygur and Han Chinese.

The present study focused solely on positive emotions. As the processing of positive emotions reflects the distinctive cultural values on emotions in Chinese culture, it is possible that Uygur Chinese could have a different regulation style and processing of negative emotions compared to Han Chinese. The processing of negative emotions could be a promising direction for future research in exploring the subcultural differences in emotion processing. Research in cultural neuroscience suggests that cultural and geographical factors could shape neural mechanisms underlying human behavior. In future studies, traditional self-report measures and neuro-scientific methods should be combined in order to improve the scope of such cultural studies.

## Ethics Statement

Informed consent was obtained before the study. The research protocol was approved by the Institutional Reviewing Board at Shenzhen University.

## Author Contributions

XD is the first and corresponding author of this paper. She made contributions to conception and design of the study, acquisition of data, analysis on data, interpretation of data, drafting the article, and finalizing the article. YY mainly contributed to the study by data collection and analyses. LS mainly contributed to the study by helping to finalize the article.

### Conflict of Interest Statement

The authors declare that the research was conducted in the absence of any commercial or financial relationships that could be construed as a potential conflict of interest.
